# Pleural infection: a retrospective study of clinical outcome and the correlation to known etiology, co-morbidity and treatment factors

**DOI:** 10.1186/s12890-018-0726-1

**Published:** 2018-10-12

**Authors:** Christian Niels Meyer, Karin Armbruster, Michael Kemp, Trine Rolighed Thomsen, Ram Benny Dessau, for The Danish Pleural Empyema group, Christian N Meyer, Thomas Ringbæk, Karin Armbruster, Torben Evald, Asbjørn Høegholm, Alan Victor, Lars C Laursen, Thyge L Nielsen, Mogens Kappel, Henriette Enevoldsen, Erik Munch, Signe Rosenlund, Jannie Broberg, Alice Friis-Møller, Michael Kemp, Ram B C Dessau, Michael Pedersen, Britta Bruun, Trine R Thomsen, Per H Nielsen

**Affiliations:** 1 Department of Internal Medicine, Zealand University Hospital Roskilde, Sygehusvej 10, 4000 Roskilde, Denmark; 2 Department of Clinical Medicine, University of Copenhagen, Copenhagen, Denmark; 3 Department of Respiratory Medicine, Copenhagen University Hospital Gentofte, Gentofte, Denmark; 4 Department of Clinical Microbiology, Odense University Hospital, University of Southern Denmark, Odense, Denmark; 5 Danish Technological Institute, Life Science, Århus and Section of Biotechnology, Aalborg University, Aalborg, Denmark; 6 Department of Clinical Microbiology, Slagelse Hospital, Slagelse, Denmark

**Keywords:** Empyema, Pleural, Infection, Pleural disease, Pyothorax, Respiratory tract infection

## Abstract

**Background:**

We explored the hypothesized importance of early knowledge of microbiological etiology in patients with pleural infection, including comorbidity and treatment factors in the outcome analyses.

**Methods:**

Data from the medical records of a large cohort of 437 consecutive patients in 9 hospitals in East-Denmark were included retrospectively.

**Results:**

Microbiology, co-morbidity, therapy and outcome are described in detail. Patient groups with microbiology negative and known bacterial etiology had a similar 30-day and 90-day mortality. There were no differences in initial antibiotic treatment regimens, antibiotic treatment duration, rate of intra-pleural fibrinolysis treatment, surgical referral rate, and ICU admittance rate. Patients with microbiology negative etiology were younger (60.8 vs 64.3 years) and fewer had predisposing risk factors (59% vs 71%), but pleural drainage was more often delayed (49% vs 36%). Mortality was similar in patients treated with either of the two nationally recommended initial antibiotic regimens. However, higher 90-day mortality (22.5% vs 9.7%), disease severity (31.5% vs 6.2%), and ICU admittance rate (21.3% vs 2.9%) was observed in a sub-group with initial broad-spectrum treatment compared to patients receiving the nationally recommended initial treatments, irrespective of knowledge of etiology. Several factors correlated independently to 90-day mortality, including age, predisposing risk factors, surgical referral (Odds-Ratios > 1), drainage delay and intra-pleural fibrinolysis (ORs < 1).

**Conclusions:**

No difference was found between patients with microbiology negative and known bacterial etiology regarding outcome or treatment parameters. Treatment factors and predisposing factors independently relating to mortality were found in the cohort. Broad-spectrum antibiotics were initially used for treatment of patients with more severe illness and poorer outcome.

## Background

Pleural bacterial infection is most often a complication to pneumonia, but with an increased morbidity and mortality. Recommended treatment consists of antibiotics and pleural drainage. Surgical therapy with thoracoscopy and debridement or decortication may be needed in a minority of patients to obtain clinical stabilization and treatment success [1]. An adequate initial antibiotic regimen takes into account the locally expected spectrum of bacterial etiology and the antibiotic susceptibility, but this specific information may not be present in up to 47% of cases with microbiology negative etiology due to recent outpatient antibiotic treatment or due to delayed in-hospital sampling [2–4]. An antibiotic treatment duration of 3–6 weeks has been recommended for pleural infection [1]. In observational studies, the presence of underlying diseases, predisposing risk factors, and treatment factors have been associated with outcome [1, 4]. In randomized controlled trials, treatment factors such as active pleural drain irrigation or intra-pleural treatment with a fibrinolytic agent plus DNase without routine use of active drain irrigation correlated to outcome [5, 6]. With a limited number of patients included at individual participating centers and not including all consecutive patients in trials, the possibility of patient selection bias cannot be excluded [6].

The aim of the study was to investigate whether patients without verified bacterial etiology differed from patients with known etiology with regard to outcome in a large cohort of consecutive patients with pleural infection. In addition, the role of predisposing risk factors, initial antibiotic treatment regimens and other treatment factors was investigated.

## Methods

### Study population

All consecutive adult patients (*n* = 437) admitted with pleural infection at nine hospitals in Eastern Denmark without an in-house thoracic surgery department in a 3.5 year period (2008–2011) were included in the Danish Pleural Empyema Project, geographically covering a population of 1.96 million adults (36% of the Danish population). No patients with pleural infection were admitted directly to the only tertiary hospital with in-house thoracic surgery in the region, as patients could only be referred from satellite hospitals as the actual practice of patient visitation. Patients were identified using three complementary strategies in order to include all patients and to counteract selection: combining the trial related prospective collection of microbiological samples with the databases of the departments of clinical microbiology, combined with the ICD-10 codes DJ860 and DJ869 in the hospital codes of diagnosis at discharge. Microbiological data was routinely collected and included microscopy, culture, and antibiotic susceptibility. The intention in the study protocol was prospectively to obtain samples from all relevant patients in the participating centers and to freeze samples for later possible bacterial identification by PCR-technology. Successful freeze samples were obtained from 72 patients of the cohort, thus adding microbiological information to few poly-microbial samples.

Clinical data included symptoms, history of predisposing risk factors, clinical findings, radiology, biochemical findings, treatment before and after hospital admission, timing of and type of therapy, length of hospital stay (LOS), intensive care unit (ICU) admittance, and all-cause mortality at day 30 and at day 90.

Two patients who were not treated with antibiotics, according to the patient’s wish or by order of the attending physician, were excluded from the study. Six patients had missing data on antibiotic treatment and were excluded.

One medical record was missing. Thirteen cases of pleural infection with attempted but unsuccessful thoracocentesis sampling and with no other positive microbiological sample were included in the analysis of the whole cohort, but were not included in statistical analyses regarding knowledge of etiology. The 13 patients with no successful pleural sample for microbiology had a trend to higher 90 day mortality (29% vs 11.7%, *p* = 0.054) but did not differ significantly in length of antibiotic treatment, proportion of nosocomial infection, pre-admission antibiotic treatment, predisposing risk factor or 30 day mortality (all *p* > 0.19), when compared to the rest of the cohort. Length of hospital stay was shorter (7.6 vs 17 days).

### Definitions

Nosocomial or hospital associated infection was defined according to the Center for Disease Control and Prevention criteria (CDC) [7].

#### Severe illness

Hypotension with systolic blood pressure < 95 mmHg, heart rate > 150 beats/min, nasal oxygen-flow above 4 L/min, other abnormities of severe sepsis, or need for ICU treatment.

#### Intra-pleural fibrinolytic treatment

The national guideline at the time from the Danish Society of Pulmonary Medicine (which had BTS guidelines as a reference) mentioned the possibility of 3 days treatment with either streptokinase or tissue plasminogen activator (t-PA). Treatment did not include DNase for any patients, as the study-period was prior to 2012, when superiority of the combination tissue plasminogen activator (t-PA) plus DNase compared to placebo was demonstrated, with a similar but non-significant difference with t-PA alone compared to placebo [6].

#### Predisposing risk factors or conditions

Alcoholism (ethanol intake > 50 g/day), cancer within the recent 5 years, hematologic malignancy, severe liver or renal disease, COPD, immune-deficiency, HIV, diabetes, aspiration tendency caused by neurologic disease, active or treated rheumatologic disease.

#### Pleural drainage

The techniques and drain types and sizes used and the type of medical persons performing the pleural drainage varied between the 9 participating centers, and at the individual center varied during the time of the day or weekends. The chosen drain size presumably varied according to the treating physician’s judgement of the individual patients need, though the national guideline suggested using a minimum 10–14 F drain. According to the national guideline, flushing of the drain with minimum 100 ml saline twice daily was recommended.

#### Delay in thoracocentesis and in pleural drainage

Number of days from detection of pleural effusion on chest X-ray or CT-scan.

#### Antibiotic regimens

In the study period, the national and regional Danish recommendations for initial treatment regimens for pleural infection (which were adopted from and inspired by the current 2003 and 2010 BTS guidelines) were cefuroxime+/− metronidazole (CEFU) or penicillin/aminopenicillin +/− metronidazole (PENI), in accordance with the national clinical microbiological findings and antibiotic susceptibility. We defined broad-spectrum treatment (BROAD) as these initial antibiotic regimens with further antibiotics added (most often a quinolone or a macrolide), or the use of other broad-spectrum regimens (most often meropenem or piperacillin/tazobactam). Each case was categorized according to the predominant antibiotic treatment (given > 50% of the time) during the first 3 days of treatment.

#### Bacterial etiology sub-groups

Cases were categorized as streptococci, *Staphylococcus aureus*, mixed infection, anaerobes only, enterobacteriaceae, other bacterial etiology, or microbiology negative etiology. This subgroup division was chosen according to our earlier publications [3, 4] and was included in the estimation and decision on sample size of the study.

### Statistical analysis

All statistical analyses were performed using Statistica 7 ® (StatSoft, Tulsa OK, USA), R-statistics (www.r-project.org), SPSS (ver 23, IBM Cooperation) or Statcalc (EpiInfo 7.2, CDC, Atlanta GA, USA). T-test or Mann-Whitney U test was used for continuous data. Chi-square or Kruskal-Wallis test was used for categorical data, backward logistic regression for multivariate outcome analyses. The individual parameters were chosen from the clinically relevant factors in the univariate analyses (from biological plausible importance and earlier publications) and the planned number of included parameters in the model according to the estimations of sample size, and estimated outcome frequency in the study design. Interaction analyses were performed as parts of the analyses. *P* < 0.05 was considered statistically significant.

## Results

The main results focus on differences in outcome or other important variables between cases with microbiology negative etiology and cases with known etiology, and are presented in Tables 1 and 2.

**Table 1:** Clinical factors, predisposing risk factors, severity factors, treatment factors, and outcome in pleural infection, in patients with microbiology negative or known etiology according to the initial antibiotic regimen

	Microbiology Negative					Known Etiology					*p* = ^§^
	PENI	CEFU	broad	All	*p* = ^*^	PENI	CEFU	broad	All	*p* = ^*^	
*n*=, (%)	62 (31)	101 (49)	37 (20)	200		57 (26)	106 (49)	49 (23)	216		
Age (years)	59.7	62.9	57.7	60.8	0.27	63.5	65.5	62.5	64.3	0.37	0.02
para-pneumonic (%)	93.5	89	92	90.5	0.90	86	90.7	85.7	88.4	0.01	0.43
Nosocomial (%)	8.0	12	13.5	10.9	0.51	10.5	20	18.4	17.2	0.24	0.06
Risk factors (%)	44^#^	68^#^	54	59	0.22	59	73	80	71	0.45	0.009
AB before hospital admittance (%)	48	57	36	50.3	0.61	39	40	51	42.5	0.44	0.13
Severely ill (%)	4.8	5.0	37	11.1	< 0.001	10.7	5.7	32	13.2	0.0001	0.90
Thoracocentesis delay, median / mean (days)	4.5 /6.3	1 /3.6	2 /2.9	2.5 /4.7	0.01	2 /4.7	1 /2.9	1 /3.4	1/3.5	0.07	0.004
AB before thoracocentesis (%)	97^#^	80^#^	91	89.1	0.37	81	75	78	76.4	0.70	0.0008
AB treatment, median days	38.5	34	34.5	35	0.48	38	39.5	36.5	38	0.49	0.30
Pleural drainage delay > 2 days (%)	57	48	40	49	0.33	45	30	39	36	0.11	0.02
Fibrinolysis intrapleural (%)	27	33	28	30	0.71	40	31	31	34	0.45	0.43
ICU admittance (%)	4.8	2.0	18.9	5.9	< 0.001	5.3	1.9	25	7.9	< 0.0001	0.45
Mortality 30-day (%)	1.6	8.0	18.9	7.9	0.025	7.0	7.5	12.2	8.8	0.55	0.76
Mortality 90-day (%)	3.2	9.9	21.6	10.3	0.03	12.3	10.4	22.4	14.4	0.12	0.21
Surgery referral (%)	8.1	14.9	10.8	14.3	0.41	15.8	7.5	20.4	14.8	0.06	0.88
LOS, median (days)	15.5	15	19.5	16	0.25	19	18	19	18	0.49	0.22

*AB* antibiotics, *ICU* intensive care unit, *LOS* length of hospital stay

^*^comparing three treatment regimens. ^§^comparing all etiology-negatives and etiology-positives

**Table 2:** Clinical factors, predisposing risk factors, severity factors, treatment factors, and outcome in the whole cohort^a^ of patients with pleural infection according to initial antibiotic regimen

	PENI	CEFU	Broad	All^a^	^*^p-value
*n*=, (%)	125 (29)	215 (50)	89 (21)	429	
Age (years)	61.4	63.9	60.7	62.6	0.12
Pneumonic (%)	90.4	89.8	88.8	89.7	0.12
Nosocomial (%)	8.8^#^	16.2^#^	15.7	14	0.09
Predisposing risk factors (%)	53.6^#^	70.7^#^	68.6	65.6	0.01
Antibiotics before hospital admittance (%)	40.8	45.6	42.7	43	0.62
Severely ill (%)	8.8	5.1	31.5	11.6	< 0.0001
Thoracocentesis delay, median /mean (days)	3 / 5.5	1 / 3.1	1 / 3.9	1 / 4.0	0.0003
Antibiotics before thoracocentesi (%)	87.2^#^	74.9^#^	77	77.6	0.24
Antibiotic treatment, median (days)	38.5	35	35.5	35	0.79
Pleural drainage delay > 2 days (%)	38.4	29.8	29.2	31.6	0.22
Fibrinolysis intrapleural (%)	32	30.7	28.1	30.2	0.96
ICU admittance (%)	4.8	1.9	21.3	6.6	< 0.0001
Mortality 30-day (%)	4.8	7.9	14.6	8.7	0.06
Mortality 90-day (%)	8	10.7	22.5	13	0.006
Surgery referral (%)	11.2	10.7	14.6	11.7	0.75
LOS, median	16	16	19	17	0.15

^a^ incl 13 patients with no successful thoracocentesis ^*^ comparing three treatment regimens. ^#^ = *p* < 0.05 for PENI vs CEFU

For the whole cohort (*n* = 429), mean age was 62.6 years (SD 14.8), 11.6% of the patients were severely ill (see Definitions) at admittance, with 6.6% admitted to the ICU. Length of ICU stay was median 6 days (range 1–25). The infection was hospital associated in 14% of cases, and 43% of the patients were treated with antibiotics before hospital admittance. Antibiotic treatment was started before thoracocentesis in 77.8% of the cases. Median duration of antibiotic treatment after hospital admission was 35 days. Intra-pleural fibrinolysis treatment was given to 30.2% of patients. Median length of hospital stay was 17 days (IQR = 11–25), 30-day mortality was 8.7%, 90-day mortality was 13% and the surgical referral rate was 11.7%.

The categorized bacterial etiology sub-groups consisted of pleural effusions with streptococci (23%), *Staphylococcus aureus* (3.7%, all were MSSA), mixed infection (11%, mostly including anaerobes), anaerobes only (1.8%), enterobacteriaceae (1.8%), other bacterial etiology (8.2%), and microbiology negative (51%).

Among the 437 consecutive patients with pleural infection, a categorized initial antibiotic regimen was administered to 200 with microbiology negative bacterial etiology, to 216 with known etiology, and to 13 with no successfully obtained pleural samples (Tables 1 and 2). The remaining eight patients could not fulfill the criteria of initial antibiotic regimen sub-groups.

### Descriptives and demographics

Patients with microbiology negative etiology were marginally younger (60.8 vs 64.3 years, *p* = 0.02), were more often treated with antibiotics prior to thoracocentesis (89.1% vs 76.4%, *p* < 0.001), and fewer had pre-defined risk factors (59% vs 71%, *p* < 0.01) compared to patients with known etiology, but had similar length of hospital stay (16 vs 18 days, *p* = 0.22) (Table 1).

The other recorded severity factors did not differ significantly between patients with microbiology negative and known bacterial etiology: The proportions of patients with severe disease, with nosocomial infection, or the proportion treated with antibiotics before hospital admission were comparable (all *p* > 0.21) (Table 1).

For the whole cohort, patients with broad-spectrum initial treatment regimens were more often severely ill (31.5% vs 6.2%) and more frequently admitted to the ICU (21.3% vs 2.9%) than patients treated with the nationally recommended initial regimens PENI or CEFU, all *p* < 0.0001 (Table 2). This was also demonstrated separately in each patient sub-category of microbiology negative or known etiology, all *p* < 0.001 (Table 1).

### Therapy

The nationally recommended initial antibiotic regimens PENI (29%) or CEFU (50%) dominated over broad spectrum therapy (21%), and the distribution of these three regimens were similar in patients with known and microbiology negative etiology, (Tables 1 and 2). Patients with microbiology negative and known etiology had a comparable median duration of antibiotic therapy (35 vs 38 days, *p* = 0.30), proportion of intrapleural fibrinolytic treatment (30% vs 34%, *p* = 0.43), and a similar proportion of surgical referral (14.3% vs 14.8%, *p* = 0.88) (Table 1). Patients with microbiology negative etiology had a longer delay until thoracocentesis (median 2.5 days vs 1 day, *p* < 0.01) and more often a delay in drainage > 2 days (49% vs 36%, *p* = 0.02).

### Outcome

The length of hospital stay (LOS), 30-day mortality, 90-day mortality, need for surgical referral, or ICU-admittance did not differ between patients with microbiology negative and known etiology (all *p* > 0.20) (Table 1).

In the univariate analyses, 30-day and 90-day mortality did not differ significantly between the six microbiological sub-groups of known etiology (7.9% vs 6.3% vs 0% vs 12.5% vs 12.5% vs 8.3% *p* = 0.85; 9.9% vs 18.8% vs 12.5% vs 14.6% vs 25% vs 19.4% *p* = 0.65). When including microbiologic sub-groups in a logistic regression model (203 cases with known etiology) with age, predisposing risk factor status, nosocomial status, pleural drain delay > 2 days, and intra-pleural fibrinolysis, the microbiologic sub-group parameter was not independently correlated to 30-day or 90-day mortality (*p* = 0.63 and, *p* = 0.90, respectively).

Comparing patients treated with the two recommended initial therapy regimens (PENI group vs CEFU group), there were no significant differences in LOS, 30-day mortality, 90-day mortality, surgical referral, or ICU admittance, neither in the whole cohort or separately in patients with microbiology negative or known etiology (all *p* > 0.11) (Tables 1 and 2).

Patients treated with a broad-spectrum initial regimen trended to have a higher 30-day (14.6% vs 6.8%, *p* = 0.06), and had a higher 90-day mortality (22.5% vs 9.7%, *p* < 0.01) compared to patients receiving PENI or CEFU, in the whole cohort (Table 2) and separately in the group with microbiology negative etiology, with a similar trend not reaching statistical significance in the group with known etiology (Table 1).

In univariate analyses, 30-day and 90-day mortality in the whole cohort (the actual direction and size of odds-ratios (OR) and the 95% confidence intervals (CI) are presented in Table 3) correlated to mean age (71 vs 61.3 years and 72 vs 61.7 years, respectively; both *p* < 0.001), pre-disposing risk factors (OR = 4.4 [1.5–12.7] and 5.7 [2.2–14.5], both *p* < 0.003), intra-pleural fibrinolysis (OR = 0.17 [0.05–0.58] and 0.35 [0.16–0.72], both *p* < 0.006). In addition, the 90-day mortality correlated to nosocomial infection (OR = 2.3 [1.16–4.5], *p* = 0.01), pleural drainage delay > 2 days (OR = 0.40 [0.20–0.83], *p* < 0.02), but did not reach significantly correlation to referral for surgery (OR = 2.0 [0.97–4.2], *p* = 0.055).

**Table 3:** Outcome analyses

	90-day mortality				30-day mortality			
	Uni-variate		Multi-variate		Uni-variate		Multi-variate	
	OR	95% CI	OR	95% CI	OR	95% CI	OR	95% CI
Age	–	–	1.06	1.04–1.09	–	–	1.06	1.03–1.10
Intrapleural fibrinolysis	0.35	0.16–0.72	0.35	0.14–0.77	0.17	0.05–0.58	0.19	0.04–0.54
Nosocomial infection	2.3	1.16–4.5			0.94	0.35–2.5		
Pleural drainage delay > 2 days	0.40	0.20–0.83	0.33	0.15–0.68	0.52	0.23–1.2	0.33	0.16–0.92
Surgery referral	2.0	0.97–4.2	3.1	1.2–7.3	0.64	0.19–2.2		
Predisposing Risk factors	5.7	2.2–14.5	4.2	1.7–12.6	4.4	1.5–12.7	3.3	1.3–11.9

The correlation of 90-day and 30-day mortality to hypothesized important categorical factors among 437 patients with pleural infection, in uni-variate and multivariate analyses

The multivariate outcome analyses included age, predisposing risk factors, nosocomial infection, pleural drainage delay > 2 days, intra-pleural fibrinolysis, and referral to surgery in the model (direction and size of ORs and CIs are presented in Table 3). The 30-day mortality correlated independently to age (OR = 1.06 [1.03–1.10], *p* < 0.0001), intra-pleural fibrinolysis (OR = 0.19 [0.04–0.54], *p* = 0.007), predisposing risk factors (OR = 3.3 [1.3–11.9], *p* = 0.03), and pleural drainage delay (OR = 0.33 [0.16–0.92], *p* = 0.04). The 90-day mortality correlated independently to age (OR = 1.06 [1.04–1.09], *p* < 0.0001), predisposing risk factors (OR = 4.2 [1.7–12.6], *p* = 0.004), referral to surgery (OR = 3.1 [1.2–7.3], p = 0.01) with ORs > 1, and pleural drainage delay (OR = 0.33 [0.15–0.68], *p* = 0.005), intra-pleural fibrinolysis (OR = 0.35 [0.14–0.77], *p* = 0.01) with OR < 1. (Table 3).

## Discussion

In this study, there were 200 or more patients with pleural infection in each of the two etiology groups, and the bacterial findings were similar to recent European studies [1].

The more severe illness and poorer outcome in the group of patients initially treated with broad-spectrum antibiotics most probably reflects clinical decisions based on the severity of the condition at admission to hospital. But because the rate of fibrinolysis or the rate of referral to surgery were not significantly different in this broad-spectrum group, the detrimental outcome may rather be due to a systemic sepsis effect of the disease.

By definition, change in antibiotic treatment within a few days according to results of susceptibility testing is not an option in cases with microbiology negative etiology and this may theoretically affect outcome. In our study, patients with microbiology negative etiology were more often treated with antibiotics before thoracocentesis, and the effect of antibiotics before microbiological sampling may account for this fact. In clinical practice, the physician’s motivation for swift pleural drainage may also depend on the presumed effusion volume and of disease severity in the individual patient; apart from the possible delay caused by lack in recognition of the condition. The slightly younger age, lower rate of predisposing risk factors (underlying diseases), and the trend towards fewer nosocomial infections in patients with microbiology negative etiology could bias prognosis towards a better outcome. On the other hand, the delay in initiating pleural drainage theoretically could result in a poorer outcome. Even though all other medical and surgical treatment measures and severity measures in this study were similar between patients with known and microbiology negative etiology, we were pleased to find that all outcome measures were comparable in the two groups. Thus, we could not confirm a hypothesized difference in outcome according to whether etiology was known or microbiologically negative.

In the literature, not many large studies (with more than 150 patients) have been published regarding pleural infection in adults, and, to our knowledge, none focused on the correlation between knowledge of etiology and clinical outcome. Compared to a large randomized controlled British study (MIST-1) [8] of intra-pleural streptokinase treatment in 430 patients with pleural infection, the patients in our cohort were of similar age (62.5 vs 60.5 years) and had similar outcomes with regard to 3 month’s mortality (13% vs 15%), referral rate for surgery (11.7% vs 15%), and LOS (17 day vs 12.5 days). In the MIST-1 trial, disease severity or outcome was not reported according to microbiology negative or known etiology, and a direct comparison to our cohort is therefore not possible [8]. The randomized MIST-2 trial cohort with 193 included patients reported a similar age (59 vs 62.5 years) and referral rate for surgery (16% vs 11.7%), but a lower 3 month’s mortality (8% vs 13%) and a similar length of hospital stay (20 vs 17 days) [6]. In the MIST-2 trial, outcomes were reported according to the four intra-pleural treatment randomization arms and were not categorized or reported according to knowledge of etiology [6]. A Danish study of 158 patients from a 9 year period just prior to our study period reported only cases of verified etiology. Compared to the present study, patients had similar age (63 years vs 62.5), a higher mortality (27% vs 13%), a longer hospital stay (29 days vs 17 days), a higher ICU-admittance rate (17% vs 6.6%) and more referrals to surgery (34% vs 11.7%) [4]. Further publications with large cohorts of patients with pleural empyema were not found for possible comparison with our study regarding the main question of disease severity or outcome according to knowledge of etiology.

The outcome analyses in our study demonstrated independent correlations between mortality and high age, referral to surgery, predisposing risk factors (OR > 1), and delayed pleural drainage more than 2 days, and intra-pleural fibrinolysis (OR < 1). Because the treatment factors were not randomized in this retrospective study, the causal relations will be rather hypothetical, despite controlling for factors included in the multivariate analysis. One can expect, that the attending physicians have judged the need for and the possibility of some interventions (f.ex. intentional or unintentional delay in drainage, referral for surgery, or intra-pleural fibrinolysis) differently according to disease acuteness, disease severity, the frailness of the patient, and the sufficient or the insufficient improvement in the individual patient. Also, lack of timely recognition of the condition may be a factor in some cases.

Regarding a possible improvement in outcome using initial treatment with early thoracoscopic debridement, a few small studies have demonstrated promising results of reduced length of hospital stay compared to initial treatment with pleural drainage [9, 10]. Larger and better powered randomized trials are needed in order to clarify the potential role of early pleural debridement in the adult population. However, early thoracoscopic debridement was not a therapeutic option in our hospitals.

In analogy with PSI, CURB-65, SOFA and other prognostic scores in pneumonia, the RAPID-score (**r**enal failure, **a**ge, non-**p**urulence, (hospital acquired) **i**nfection source, **d**ietary factors (=low albumin)) was recently developed as a prognostic score in patients with pleural infection and includes 5 non-interventional factors [11]. The renal parameter used (blood urea nitrogen) was not routinely assessed at admission to our hospitals, and thus, the RAPID-score could not be evaluated in our cohort. In our study-cohort, we did find association of mortality to the available RAPID parameters age and nosocomial infection, but we did not separate out renal insufficiency or malnutrition as individual factors (from the predisposing factors) in our multi-variate analyses. Recently, the RAPID-score was validated in a not very typical study-cohort of all ages with a large number of *Staphylococcus aureus* infections (40%) and nosocomial infections (41%) among 187 culture-positive patients, assessing stratified mortality risk at 3 months, but also at 1 year, 3 year and 5 year [12]. The stratification power was more pronounced at 3 months and in the sub-group of patients treated with invasive surgery [12].

The main strengths of our study are the sample size and the completeness of the cohort using several strategies for patient inclusion. Although we used the databases of clinical microbiology and the national clinical diagnosis register, and attempted prospective inclusion, we cannot rule out the possibility of missing cases due to mislabeling in the national clinical diagnosis register in culture-negative cases of pleural infection. Our study has inborn limitations (including some missing data) due to the predominantly retrospective mode of data collection.

As the occurrence of the time-dependent covariates (f.ex. delayed drainage > 2 days, referral to surgery) are possible always in the surviving sub-group but may only be possible in some patients in the dying group (if the intended intervention is not delayed before the outcome of death), the immortal time bias is a possible limitation in the logistic regression mortality analysis. Among the 56 dying patients in our cohort with four patients dying within 2 days, two were drained and the two others were left to palliative care without drainage. The referral to surgery factor was not documented as an unfulfilled intention in the medical charts in any of the dying patients, but missing documentation cannot be ruled out. Thus, we suggest that the covariate delayed drainage > 2 days did not suffer from immortal time bias of clinical importance. A potential shortcoming of the analysis in our database is that the possible immortal time bias could not be estimated for the covariate referral to surgery, and therefore effect estimates of referral to surgery should be interpreted with caution.

## Conclusion

This study adds outcome results, clinical data and treatment information regarding the serious and often complicated condition of pleural infection from a large patient cohort. Comparing patients with microbiology negative and known bacterial etiology, no differences were found in observed mortalities, in underlying disease severity, initial antibiotic treatment type, total treatment duration, or in the rate of surgical referral. Thus, lack of data on etiology and antibiotic susceptibility in many of the patients did not signal a change in outcome in a geographical area with expectedly well-functioning health-care facilities and limited problems of antibiotic resistance, though the limitations of a retrospective design precludes from conclusions on causation.

## Abbreviations

AB: Antibiotic
CDC: Center for Disease Control and Prevention
CEFU: Cefuroxime +/− metronidazole
COPD: Chronic obstructive pulmonary disease
DNA: Deoxyribonucleic acid
ICD: International Classification of Diseases
ICU: Intensive care unit
LOS: Length of hospital stay
OR: Odds ratio
PCR: Polymerase chain reaction
PENI: Penicillin/aminopenicillin +/− metronidazole
t-PA: Tissue plasminogen activator
